# Case Report: Tumor Microenvironment Characteristics in a Patient With HER2 Mutant Lung Squamous Cell Carcinoma Harboring High PD-L1 Expression Who Presented Hyperprogressive Disease

**DOI:** 10.3389/fonc.2021.760703

**Published:** 2021-12-24

**Authors:** Lixia Xia, Yinghui Yu, Fen Lan, Junrong Yan, Jinfan Li, Wen Li, Yang Xia

**Affiliations:** ^1^ Key Laboratory of Respiratory Disease of Zhejiang Province, Department of Respiratory and Critical Care Medicine, Second Affiliated Hospital of Zhejiang University School of Medicine, Hangzhou, China; ^2^ Medical Department, Nanjing Geneseeq Technology Inc., Nanjing, Jiangsu, China; ^3^ Department of Pathology, Second Affiliated Hospital of Zhejiang University School of Medicine, Hangzhou, China

**Keywords:** hyperprogressive disease, non-small cell lung cancer, PD-1 inhibitor, HER2, macrophage

## Abstract

**Background:**

High PD-L1 expression in non-small cell lung cancer (NSCLC) is evident to predict elevated immunotherapy efficacy, to which NSCLC with onco-driver gene mutations is probed with poor responsiveness. Thus, it is of great interest to investigate how effective immune monotherapy is in the presence of concurrent high PD-L1 expression and driving gene mutation.

**Patients and methods:**

We present a case of squamous lung cancer with high PD-L1 expression and HER2 exon 20 insertion (20Ins) who presented hyperprogressive disease (HPD) after being treated with PD-1 inhibitor.

**Results:**

A 71-year-old female was diagnosed with advanced squamous lung cancer with 98% tumor proportion score of PD-1 and 20ins. She benefited from first-line docetaxel cisplatin followed by 2 months second-line afatinib. Third-line pembrolizumab monotherapy was then given. Unfortunately, she rapidly progressed with dramatically enlarged primary site as well as mediastinal lymph nodes and pleural effusion only 2 weeks later, presenting severe dyspnea and dysphagia. Re-biopsy was conducted, and we found that compared with the baseline, CD8+ T cells were largely recruited only in tumor stroma but not in tumor parenchyma. Tumor-associated macrophages were notably increased in both tumor stroma and parenchyma. Concomitantly, CD56dim NK cells in tumor parenchyma were decreased.

**Conclusions:**

Application of immune monotherapy in patients with positive driver genes demands extreme caution, even harboring high PD-L1 expression. Abnormality of tumor microenvironment might be critically involved in immune checkpoint inhibitor-induced HPD. Further study in greater depth is required.

## Introduction

ICI-based modalities have become the standard first-line treatment for advanced NSCLC. PD-L1 expression is the most widely used biomarker to identify the potential beneficiaries and pembrolizumab is currently approved in the U.S. for the first-line treatment of advanced NSCLC with PD-L1 TPS of 1% or more. Also, ICIs demonstrate benefit over chemotherapy despite PD-L1 expression levels in second and further lines. However, the therapeutic role of ICIs in oncogene-driven NSCLC remains unsatisfied. HER2 mutations have been identified as emerging and uncommon oncogenic driver in NSCLC. To date, there is no standard targeted therapy for this subgroup of patients (1). Here, we report a case of hyperprogressive disease (HPD) in squamous lung cancer carrying high PD-L1 expression and HER2 exon 20 insertion mutation treated with PD-1 inhibitor monotherapy as posterior line therapy, and we depicted the tumor microenvironment before and after PD-1 inhibitor, aiming to figure out the possible mechanisms of HPD.

## Case Report

**Figure 1:**
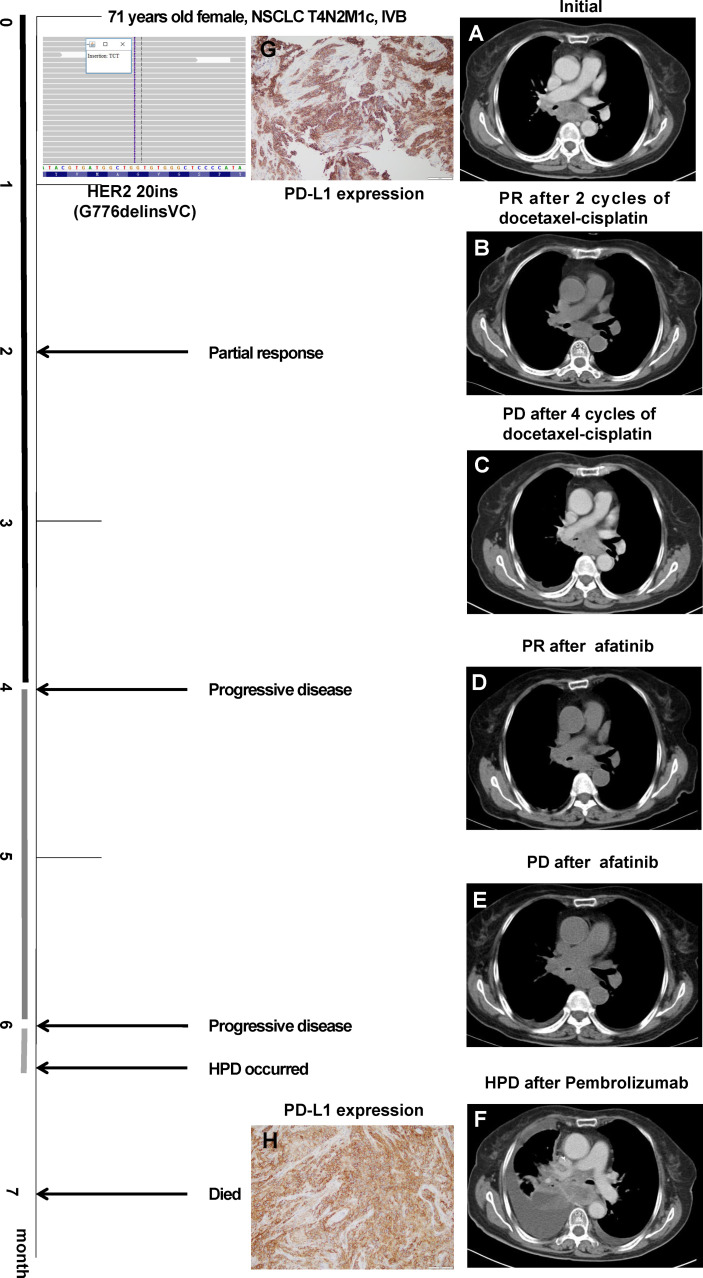
Chest CT images throughout the disease course. **(A)** Initial evaluation showed enlarged mediastinal lymph node. **(B)** Partial response (PR) after docetaxel-cisplatin. **(C)** Progressive disease after four cycles of docetaxel-cisplatin. **(D)** PR after afatinib. **(E)** Progressive disease after 2 months of afatinib. **(F)** Hyperprogressive disease after one cycle of pembrolizumab. PD-L1 expression before **(G)** and after **(H)** pembrolizumab monotherapy.

**Figure 2:**
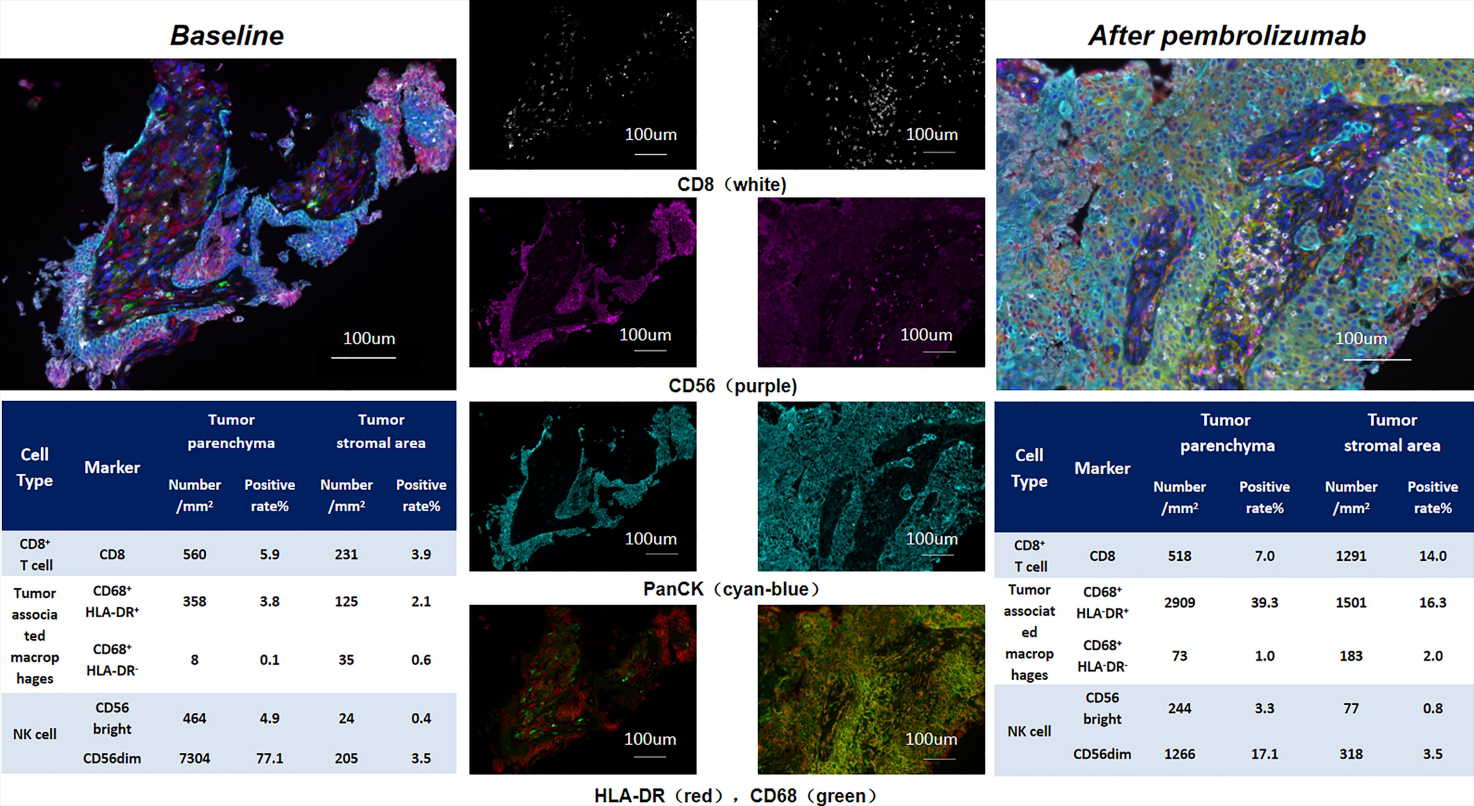
Tumor mircoenvironmental features before and after pembrolizumab treatment.

A 71-year-old female without smoking history underwent cough for over 2 months. Chest CT scan (**Figure 1**) showed right lower lung mass with multiple mediastinal lymph nodes enlargement. Pathological findings of the mass showed poorly differentiated lung squamous cell carcinoma. Immunohistochemical staining showed TTF-1 (–), Napsin A (–), CK5/6 (+), P63 (+), P40 (+), and CK7(+). Systemic evaluation revealed multiple bone metastasis and the TNM staging was T4N2M1c, IVB. Tissue DNA of primary site was processed and 437 genes were sequenced in the NGS platform Illumina Nextseq 500 to >500× coverage as described before (2). Targeted next-generation sequencing detected HER2 exon 20 insertion (20ins) mutation (G776delinsVC) and PD-L1 was strongly expressed (Dako-22C3, TPS 98%). She benefitted from 4 cycles of first-line docetaxel-cisplatin with progression-free survival (PFS) of 4 months, and second-line afatinib was then used signing the informed consent, where the physician introduced the indications and recommendations of afatinib in the treatment of HER2 mutant NSCLC. Although the lesion dramatically regressed, unfortunately, her tumor progressed only 2 months later. Pembrolizumab monotherapy was given. However, she developed sudden onset of severe dyspnea and dysphagia 2 weeks after the first dose of pembrolizumab. Chest CT (**Figure 1**) revealed that the tumor rapidly progressed with massive pleural effusion and significantly enlarged mediastinal lymph nodes. HPD occurred and the re-biopsy at right main bronchus showed squamous lung cancer with high PD-L1 expression (Dako-22C3, TPS 95%) and HER2 20ins (G776delinsVC). Immunohistochemical staining showed P40 (+), CK7 (+), CK20 (–), TTF-1 (–), and P63 (+). She eventually died of respiratory failure after 1 month. In order to figure out the potential mechanism of HPD, we performed multiple fluorescent immunohistochemistry analysis on the samples before and after pembrolizumab. We marked CD8, CD56, CD68, HLA-DR, and panCK, and quite interestingly, after pembrolizumab, CD8^+^ T cells were recruited only in tumor stroma without infiltrating tumor parenchyma. Concomitantly, tumor-associated macrophages were dramatically elevated; by contrast, CD56^dim^ NK cells in tumor parenchyma were decreased (**Figure 2**).

## Discussion

Squamous cell lung cancer is a highly heterogeneous disease with a wide range of mutations including TP53, PIK3CA, CDKN2A, SOX2, CCND2, NOTCH1/2, MET, and FGFR1. However, targetable onco-driver gene mutation in SCC is rare. The human ErbB family is composed of four members: EGFR (ErbB1/human epidermal growth factor receptor, HER1), ErbB2 (HER2/NEU), ErbB3 (HER3), and ErbB4 (HER4). Very recently, our group reported a cohort of SCC having EGFR mutation, who presented blunt responsiveness to EGFR-TKI while exhibiting identical mutation patterns compared with EGFR-mutant lung adenocarcinoma. In-frame insertions in exon 20 of HER2 kinase domain was the most common HER2 mutation subtype, including A775_G776insYVMA (42%), G778_P780dup (19%), and G776delinsVC (8%). In contrast to breast cancer, HER2 mutation, rather than HER2 overexpression/amplification, is the most clinically relevant HER2 aberration in HER2-directed therapies for NSCLC. To date, several HER2-targeted agents such as afatinib, pyrotinib, poziotinib, trastuzumab emtansine, and trastuzumab deruxtecan have been found to be effective in NSCLC with HER2 mutation (1). Our meta-analysis showed that afatinib monotherapy demonstrated frustrating anti-tumor activity; however, the patients harboring A775-G776ins YVMA mutation derived greater clinical benefit (3). Our case, in concert with the observation from EGFR-mutant SCC and the conclusion from meta-analysis, conferred sensitivity to afatinib, but the duration of response was rather limited.

Not only the data from Checkmate057 (4) and Atlantic trial (5) in advanced NSCLC, but also the results derived from IMpower010 (6) in an adjuvant setting, PACIFIC trial (7) in a locally advanced setting, and IMMUNOTARGET registry (8) from the real-world evidence showed blunted clinical activity of ICI in lung cancer harbored oncogenic driver alterations. Until now, there is no standard of care for HER2-mutated NSCLC. In the real clinical scenario, treatment varies from chemotherapy to targeted therapy, even immunotherapy. With respect to the role of immunotherapy in HER2-mutant NSCLC, several retrospective studies reported the outcomes. The overall response rate ranged from 12% to 27.3%, median PFS was 2.3–4 months, and median overall survival was 10.4–20.4months, which seems comparable to that of the whole population (9). Our reported patient received pembrolizumab monotherapy after afatinib resistance. Unfortunately, even with TPS as high as 95%, she developed HPD after only one cycle of pembrolizumab, although studies had shown that pembrolizumab monotherapy provides a long-term OS benefit for metastatic NSCLC with PD-L1 ≥ 50%.

HPD is a knotty pattern of progression described in cancer patients treated with ICIs with cumulated concerns. Different studies employ varied definitions of HPD. Kato (10) defined hyperprogression as time-to-treatment failure (TTF) < 2 months, >50% increase in tumor burden compared with pre-immunotherapy imaging, and >2-fold increase in progression pace. Previous studies reported the association between HPD and clinicopathological features, such as older age (>65 years), female, neutrophil-to-lymphocyte ratio (11), poor ECOG PS, mouse double minute 2/4 homolog (MDM2/4), and EGFR mutations. However, the exact mechanism for HPD remains unclear. Innate and adaptive immune systems might both play a role. Potential explanations include oncogenic signaling activation, upregulation of alternative immune checkpoints, or modulation of other protumor immune subsets (12). The evaluation of infiltrating macrophages in tumors, referred to as tumor-associated macrophage (TAM), has been well-documented to be the key component of the tumor microenvironment that influence tumor growth and progression (13). TAMs can be polarized into different phenotypes, including tumor-inhibiting M1 macrophages and tumor-promoting M2 macrophages. The stromal macrophages, which were primarily M2 TAMs, were associated with a poor prognosis in NSCLC (14). Tumor cells release various chemokines to attract macrophages, as well as other inflammatory cells, into the tumor stroma, and several substances secreted by TAMs may stimulate the proliferation and metastasis of tumor cells. Shumei Kato (15) revealed a high expression of the macrophage-associated markers CD68, which were associated with significantly worse PFS after anti-PD-1/PD-L1-based therapies. Lo Russo (16) revealed that M2-like CD163^+^ CD33^+^ PD-L1^+^ tumor-associated macrophages can block anti-PD-1 antibody functional activity by interacting with the Fc domain of the antibody. M2 TAMs may induce tumor cell aggressiveness and proliferation and increase metastatic potential, resulting in a poor prognosis in patients with NSCLC. Here, we found that CD8^+^ T cells were largely recruited in stroma region but not actively recruited in the tumor parenchyma. Accumulated lines of evidence have shown several immune-excluded phenotypes, one of which was characterized by the presence of large numbers of immune cells; however, these immune cells are located in the stroma surrounding the tumor nests without infiltrating tumor parenchyma (17, 18). Thus, the therapeutic response to ICI was blunted. The concept of immune-excluded phenotype tumor fits our observation that our patient poorly responded to ICI treatment. Such immune microenvironment can be seen in many studies regardless of mutation status; hence, we speculate that it is not HER2-specific. Moreover, we also observed increased M2 macrophages and decreased CD56^dim^ NK cells, both of which may contribute significantly to tumor growth and metastasis, in turn contributing to HPD. Of note, as the retrospective nature of our study, we are unlikely to detect inflammatory factors and chemokines; otherwise, we could provide more favorable evidence. In addition, chemotherapy and targeted therapy between the biopsies were administered; we could not exclude that some microenvironment changes may have occurred during these treatments.

Collectively, application of immune monotherapy in patients with positive driver genes demands extreme caution, even harboring high PD-L1 expression. Abnormality of tumor microenvironment might be critically involved in immune checkpoint inhibitor-induced HPD. A much deeper understanding of mechanisms leading to a tolerogenic tumor immune microenvironment in NSCLC is needed.

## Data Availability Statement

The data presented in the study are deposited in the National Genomics Data Center (NGDC) repository (https://ngdc.cncb.ac.cn/gsa-human), accession number HRA001277.

## Ethics Statement

The studies involving human participants were reviewed and approved by the Human Research Ethics Committee of the Second Affiliated Hospital of Zhejiang University School of Medicine. The patients/participants provided their written informed consent to participate in this study.

## Author Contributions

Conceptualization: YX, WL, and JFL. Data Curation: LXX, FL, YHY, and JFL. Writing—Original Draft: LXX, FL, and JRY. Writing—Review and Editing: YX, WL, YHY, and JRY. Supervision: YX, WFLU, aNndD JFINL. GAll authors contributed to the article and approved the submitted version.

## Funding

This work was supported by the Zhejiang Provincial Natural Science Foundation [LY20H010004], the National Natural Science Foundation of China [81870022], and the National Natural Science Foundation of China [81800007].

## Conflict of Interest

Author JY was employed by company Nanjing Geneseeq Technology Inc.

The remaining authors declare that the research was conducted in the absence of any commercial or financial relationships that could be construed as a potential conflict of interest.

## Publisher’s Note

All claims expressed in this article are solely those of the authors and do not necessarily represent those of their affiliated organizations, or those of the publisher, the editors and the reviewers. Any product that may be evaluated in this article, or claim that may be made by its manufacturer, is not guaranteed or endorsed by the publisher.
